# Diet composition, adherence to calorie restriction, and cardiometabolic disease risk modification

**DOI:** 10.1111/acel.14018

**Published:** 2023-10-24

**Authors:** Sai Krupa Das, Rachel E. Silver, Alistair Senior, Cheryl H. Gilhooly, Manjushri Bhapkar, David Le Couteur

**Affiliations:** ^1^ Jean Mayer USDA Human Nutrition Research Center on Aging at Tufts University Boston Massachusetts USA; ^2^ Charles Perkins Centre University of Sydney Sydney New South Wales Australia; ^3^ School of Life and Environmental Sciences University of Sydney Sydney New South Wales Australia; ^4^ Sydney Precision Data Science Centre University of Sydney Sydney New South Wales Australia; ^5^ Duke Clinical Research Institute Duke University School of Medicine Durham North Carolina USA; ^6^ Centre for Education and Research on Ageing Concord RG Hospital Concord New South Wales Australia; ^7^ ANZAC Research Institute Sydney New South Wales Australia

**Keywords:** aging, calorie restriction, human, molecular biology of aging

## Abstract

Calorie restriction (CR) is a promising approach for attenuating the risk of age‐related disease. However, the role of diet composition on adherence to CR and the effects of CR on cardiometabolic markers of healthspan remains unknown. We used the Geometric Framework for Nutrition approach to examine the association between macronutrient composition and CR adherence during the 2‐year CALERIE trial. Adult participants without obesity were randomized to a 25% CR intervention or an ad libitum intake control. Correlations of cardiometabolic risk factors with macronutrient composition and standard dietary pattern indices [Alternate Mediterranean Diet Index (aMED), Dietary Inflammatory Index (DII), and Healthy Eating Index (HEI)] were also evaluated by Spearman's correlation at each time point. The mean age was 38.1 ± 7.2 years at baseline and the mean BMI was 25.1 ± 1.7. The study population was 70% female. The CR group, but not the control, consumed a higher percentage reported energy intake from protein and carbohydrate and lower fat at 12 months compared to baseline; comparable results were observed at 24 months. Protein in the background of higher carbohydrate intake was associated with greater adherence at 24 months. There was no correlation between macronutrient composition and cardiometabolic risk factors in the CR group. However, statistically significant correlations were observed for the DII and HEI. These findings suggest that individual self‐selected macronutrients have an interactive but not independent role in CR adherence. Additional research is required to examine the impact of varying macronutrient compositions on adherence to CR and resultant modification to cardiometabolic risk factors.

## Graphical abstract

Healthy adult participants without obesity who received a 2‐year calorie restriction (CR) intervention consumed a higher percentage of energy from protein and carbohydrate and lower fat after 1 and 2 years of CR compared to baseline. Protein in the background of higher carbohydrate intake, but not lower carbohydrate intake, was associated with greater adherence to CR after 2 years. Macronutrient composition was not associated with cardiometabolic risk factors in the CR or control groups.

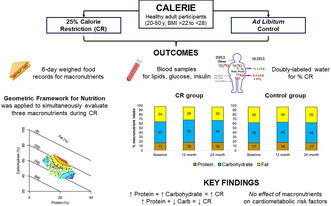

**Abbreviations**
AIC: Akaike information criterion
AL: ad libitum
aMED: Alternate Mediterranean Diet index
BMI: body mass index
BP: blood pressure
CALERIE: Comprehensive Assessment of Long‐term Effects of Reducing Intake of Energy
CR: calorie restriction
CRP: C‐reactive protein
DII: Dietary Inflammatory Index
FBG: fasting blood glucose
GFN: Geometric Framework for Nutrition
HDL: high‐density lipoprotein
HEI: Healthy Eating Index
ITT: intention to treat
LDL: low‐density lipoprotein
MR‐GLMMs: multi‐response generalized linear mixed models
MSS: metabolic syndrome score
NDSR: Nutrition Data System for Research
NIH: National Institutes of Health
RMTs: right‐angle mixture triangles
TG: triglycerides
WC: waist circumference

## 1 INTRODUCTION

The population of older adults is growing at an unprecedented rate and is paralleled by an increasing prevalence of chronic disease (Kennedy et al., 2014; Lutz et al., 2008). Aging is associated with impaired metabolic regulation and increased risk of chronic disease (Finkel, 2015), and effective strategies targeting the attenuation of age‐related disorders, particularly cardiometabolic disease and its associated disability, are therefore urgently needed (Espeland et al., 2017). Nutritional modulation of aging, in particular calorie restriction (CR), is a recognized approach for delaying the onset and progression of age‐related disease in various model organisms (Fontana et al., 2010). However, evidence in animal models suggests that the benefits of CR cannot be attributed solely to a reduction in calories. Rather, the ratio of dietary macronutrients also plays an important role in reducing age‐related disease (Bruce et al., 2013; Ingram & de Cabo, 2017; Iwasaki et al., 1988; Mair et al., 2005; Rizza et al., 2014; Solon‐Biet et al., 2014; Tatar et al., 2014; Zimmerman et al., 2003). Notably, studies that use the geometric framework for nutrition (GFN), a state‐of‐the‐art analytical approach that can simultaneously evaluate multiple dietary components rather than single nutrients and determine their associations with disease risk, support the finding that the ratio of macronutrients in the diet plays an important and independent role in influencing health (Lee et al., 2008; Senior et al., 2019; Solon‐Biet et al., 2014). However, whether self‐selected diet composition changes during CR, has not been fully explored in well‐designed human CR trials with rigorous dietary intake data and in a population without obesity. Furthermore, the question of whether these dietary changes may influence the ability to sustain CR or promote cardiometabolic risk factor modification is unclear.

The comprehensive assessment of long‐term effects of reducing intake of energy (CALERIE) study, funded by the National Institutes of Health (NIH), was the first clinical trial to demonstrate the feasibility of achieving sustained, moderate CR in healthy adults without obesity (Rochon et al., 2011). The CALERIE trial randomized participants to a CR prescription or ad libitum intake (AL) and found that 2 years of CR led to significant reductions in cardiometabolic risk factors (Das et al., 2017; Ravussin et al., 2015). However, CR participants—who aimed to reduce their baseline energy intake by 25% while self‐selecting their diet—demonstrated notable variability in adherence to CR with some participants achieving up to 30% CR. These findings raise the question of whether specific dietary components facilitate adherence and modulate responses to CR.

The current study uses the Geometric Framework for Nutrition (GFN) analytical approach to evaluate the effect of dietary macronutrient composition on adherence to CR and resultant modification to cardiometabolic risk factors in a secondary analysis of the CALERIE trial. Our primary objective was to evaluate whether self‐selected diet composition changes during CR and whether these changes predict short‐term (12 months) or long‐term (24 months) adherence to CR. Our secondary objective was to explore the relationships between macronutrient composition, standard dietary pattern scores, and cardiometabolic risk factors.

## 2 METHODS

### 2.1 Study design and data sources

CALERIE was a phase 2, multicenter, randomized controlled trial funded by the NIH and was the first to explore the translatability of CR to human aging. The study design and intervention design are described elsewhere (Rickman et al., 2011; Rochon et al., 2011). Briefly, CALERIE participants were healthy young‐ and middle‐aged adults (21–50 years) without obesity [body mass index (BMI), 22–27.9 kg/m^2^] who were randomized in a 2:1 ratio in favor of 25% reduction in caloric intake (CR group) or AL caloric intake (control group) for 2 years. Participants self‐selected their foods and were counseled to consume a nutritionally adequate diet (Rickman et al., 2011). No specific dietary pattern was prescribed. Assessments were made at baseline, 12‐ and 24‐month follow‐up for both groups. The study protocol was approved by the Institutional Review Board of each participating site. Written informed consent was obtained from all participants. The CALERIE trial is registered at clinicaltrials.gov (NCT00427193).

This secondary analysis used publicly available, de‐identified data from the CALERIE study data repository (https://calerie.duke.edu/). The data repository includes health, dietary, and cardiometabolic risk measures collected during the 2‐year trial. There is no identifiable link that can be traced to the individual participants. All CALERIE data contain unique participant identifiers that allow multiple datasets to be linked by visit to the individual participant. The CALERIE data were collected using standardized procedures, and the study data were subject to rigorous quality control prior to posting for public access.

### 2.2 Exposure measurements

Dietary data from the CALERIE study were captured by detailed 6‐day food records obtained prior to the intervention and at 12‐ and 24‐month follow‐up. Both weekdays and weekend days were included in the 6‐day food record. Nutrient information was analyzed using 2010 Nutrition Data System for Research (NDSR) software, developed by the University of Minnesota. Dietary data quality was tested using the available doubly labeled water measurements. The mean and median difference between measured and reported energy intake was within ±20% for all time points, indicating the high quality of the CALERIE dietary data and all participants were included in this analysis. Self‐reported macronutrient consumption (i.e., carbohydrate, fat, and protein) were defined as the percentage of total energy intake at each study visit. The alternate Mediterranean diet score (aMED), Dietary Inflammatory Index (DII), and Healthy Eating Index (HEI) were calculated using standard methods as described below.

The aMED score is a nine‐component, validated score that is used to estimate adherence to the Mediterranean diet in the US population (Fung et al., 2009). It is a modified version of the original Mediterranean diet score and more representative of a US diet. The nine components of the aMED score are vegetables (excluding potatoes), fruit, legumes, whole grains, nuts, fish, red and processed meats, alcohol, and the ratio of monounsaturated fat to saturated fat. The daily intake of each component was calculated as the number of servings consumed per day. For each component hypothesized to benefit health, one point was assigned for intake above the sex‐specific median. For alcohol, one point was assigned for mild to moderate consumption. For components presumed to be detrimental to health, one point was assigned for intake below the sex‐specific median. Scores for each component were summed to calculate the total aMED score for each participant and ranged from zero (non‐adherence) to nine (perfect adherence).

For the DII, scores were calculated at each study visit using methods previously described (Shivappa et al., 2014). Briefly, the DII was derived based on robust estimates of global dietary intake. Z‐scores of intake relative to the global mean were calculated. Centered percentiles for each food parameter are calculated and multiplied by the parameter‐specific inflammatory effect score. The DII scores for 28 of 45 food parameters hypothesized to affect six inflammatory biomarkers are summed to calculate an overall DII score for each individual (theoretical range: −8 to +8). A positive score indicates a pro‐inflammatory diet (i.e., less healthy), and a negative score indicates an anti‐inflammatory diet (i.e., more healthy).

The Healthy Eating Index evaluates how well dietary patterns align with the recommendations proposed in Dietary Guidelines for Americans, and the HEI‐2015 was applied to quantify adherence to the 2015–2020 Dietary Guidelines (Krebs‐Smith et al., 2018). Briefly, the HEI is comprised by 13 components reflecting different food groups and key recommendations, which are summed to obtain the total HEI score. These components include adequate intake of total fruits (maximum points: 5), whole fruits (5), total vegetables (5), greens and beans (5), whole grains (10), dairy (10), total protein (5), seafood and plant protein (5), and fatty acids (10), with moderate intake of refined grains (10), sodium (10), added sugars (10), and saturated fat (10). Intakes between the minimum and maximum standards for each component were scored proportionately. Each component is scored per 1000 kcal, with the exception of the fatty acid component as it is a ratio of unsaturated to saturated fatty acids. Scores range from zero (indicating no adherence to the Dietary Guidelines) to 100 (indicating complete adherence to the Dietary Guidelines).

### 2.3 Outcome measurements

The primary outcome of interest in this analysis was adherence to the CR intervention. Energy expenditure was measured by doubly labeled water, an objective biomarker of energy intake (Wong et al., 2014) and used to calculate percent CR attained throughout the study (Racette et al., 2012). Adherence was calculated as the natural log of the ratio of prescribed energy intake to total energy intake. An adherence value equal to 0 indicated that the participant met the energy intake prescribed by the intervention, whereas adherence less than 0 indicated that a participant consumed more energy than prescribed and adherence greater than 0 indicated that a participant consumed less energy than prescribed. Prescribed values were determined relative to baseline energy intake. Short‐ and long‐term adherence to CR were defined as adherence at 12 and 24 months, respectively.

Cardiometabolic risk factor measurements were assessed as secondary outcomes of interest and included C‐reactive protein (CRP), high‐density lipoprotein (HDL), low‐density lipoprotein (LDL), triglycerides, blood pressure, fasting glucose and insulin levels, and a metabolic syndrome score (MSS). Sex‐specific metabolic syndrome scores were computed in the CALERIE trial using mean BP (MBP = [2 × diastolic BP + systolic BP] / 3), HDL, triglycerides (TG), waist circumference (WC), fasting blood glucose (FBG), and standard deviation (SD), as detailed previously (Bateman et al., 2011; Kraus et al., 2019; Matthews et al., 1985) and outlined below:

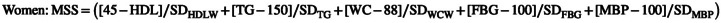

$$\mathrm{Men}:\mathrm{MSS}=\left(\left[40-\mathrm{HDL}\right]/{\mathrm{SD}}_{\text{HDLM}}+\left[\mathrm{TG}-150\right]/{\mathrm{SD}}_{\mathrm{TG}}+\left[\mathrm{WC}-102\right]/{\mathrm{SD}}_{\mathrm{WCM}}+\left[\mathrm{FBG}-100\right]/{\mathrm{SD}}_{\mathrm{FBG}}+\left[\mathrm{MBP}-100\right]/{\mathrm{SD}}_{\mathrm{MBP}}\right)$$

### 2.4 Statistical analysis

All analyses were intention‐to‐treat (ITT). Pre‐intervention demographic, anthropometric, dietary, and cardiometabolic risk factor measurements were compared for the CR and control groups.

The mean percentage macronutrient intake was calculated for each group at baseline, 12 and 24 months. Data and results were visualized using right‐angle mixture triangles (RMTs), the primary tool that has emerged for displaying compositional dietary data in the GFN (Raubenheimer, 2011) Briefly, RMTs depict a 3D diet composition (i.e., where the three components sum to 100) in a 2D Cartesian space, with the percentage of the diet coming from two nutrients on the *X* and *Y* axes, with the third nutrient captured implicitly.

To visualize how self‐selected dietary composition for each macronutrient changed over time in the CR and control groups, we depict diet composition at baseline, 12 and 24 months as their mean and 95% confidence intervals on RMTs, as well as calculating changes and presenting mean changes in the % from each macronutrient from baseline. To assess whether differences in the macronutrient composition of diet over time, and between groups were statistically significant we used a Bayesian multi‐response generalized linear mixed models (MR‐GLMMs; MCMCglmm package; Hadfield, 2010 model implemented with 500 K iterations). Here, the response variables were the logit transformed proportions of each macronutrient, and the time of measurement (baseline, 12 and 24 months) as well as group were fixed predictors, while the ID of the participant was treated as a random effect. The multiresponse approach was used as it allows us to model the inherent covariance between compositions, which are constrained to sum to 100. Effects were considered statistically significant when the estimated difference has a 95% credible interval that did not include zero.

The effects of self‐selected dietary composition on adherence to CR and cardiometabolic risk factors at 12 and 24 months were evaluated in the CR group only. We used the surface‐based approach common in the GFN, where the outcomes (e.g., adherence) are mapped as response surfaces to the nutritional space of the RMT (Saner et al., 2020). To create the surfaces for each outcome of interest, we fitted a series of three mixture models (a.k.a., Scheffes polynomials; Lawson & Willden, 2016) and selected the best fitting model by Akaike information criterion (AIC; i.e., the simplest model within 2 AIC points of the minimum AIC). Model 1 comprised a null model, which assumes no effect of diet composition, and model 2 is equivalent to the “partition” substitution model commonly used in nutritional epidemiology and assumes a linear effect of macronutrients (Saner et al., 2020; Song & Giovannucci, 2018). Finally, model 3 was a quadratic model that allows for nonlinear effects of nutrients. Where model 1 (the null model) was favored, we infer no effect of macronutrient composition on the outcome. Where either model 2 or 3 was favored by AIC, we conclude that there was an effect of macronutrient composition on the outcome of interest. All models were corrected for age and sex using the residual method. Model predictions from the AIC‐favored model were then visualized using RMTs. Finally, univariate correlations between dietary composition and dietary pattern indices with cardiometabolic risk factors were assessed by Spearman's rank correlation.

## 3 RESULTS

### 3.1 Participants

Baseline demographic, anthropometric, dietary, and cardiometabolic characteristics of the CR and control groups are shown in Table 1. Among participants with baseline data, there were 143 participants randomized to the CR intervention and 75 participants randomized to the ad libitum control group. The mean age was 38.1 ± 7.2 years at baseline and the mean BMI was 25.1 ± 1.7. The study population was 70% female. All other demographic and anthropometric characteristics were evenly distributed between the two groups.

**TABLE 1:** Baseline demographic, anthropometric, dietary, and cardiometabolic characteristics of all *CALERIE* trial enrollees by randomized intervention group.

	Total (*n* = 218)^a^	Calorie restriction (*n* = 143)	Ad libitum control (*n* = 75)
*Demographic*			
Age (years)	38.1 ± 7.2	38.2 ± 7.3	38.1 ± 6.9
Sex			
Male	66 (30.3)	44 (30.8)	22 (29.3)
Female	152 (69.7)	99 (69.2)	53 (70.7)
Ethnicity			
Non‐Hispanic	209 (95.9)	138 (96.5)	71 (94.7)
Hispanic	7 (3.2)	3 (2.1)	4 (5.3)
Unknown	2 (0.9)	2 (1.4)	0 (0.0)
Education			
Less than college	39 (17.9)	23 (16.1)	16 (21.3)
College degree	103 (47.3)	67 (46.9)	36 (48.0)
Graduate degree	76 (34.9)	53 (37.1)	23 (30.7)
*Anthropometric*			
Weight (kilograms)	71.8 ± 9.2	72.0 ± 9.5	71.5 ± 8.6
Body mass index (kg/m^2^)	25.1 ± 1.7	25.2 ± 1.8	25.1 ± 1.6
Body fat (%)	33.1 ± 6.2	32.9 ± 6.1	33.6 ± 6.6
*Dietary*			
Calories from alcohol (%)	3.2 ± 3.6	3.2 ± 3.5	3.1 ± 3.8
Calories from carbohydrate (%)	46.2 ± 6.5	46.8 ± 6.5	45.1 ± 6.3
Calories from fat (%)	33.9 ± 5.0	33.5 ± 4.9	34.7 ± 5.1
Calories from protein (%)	16.8 ± 3.2	16.6 ± 3.0	17.2 ± 3.5
Alternate Mediterranean Diet Index	4.3 ± 1.8	4.4 ± 1.8	4.0 ± 1.9
Dietary Inflammatory Index	−0.15 ± 1.8	−0.29 ± 1.84	0.10 ± 1.71
Healthy Eating Index	59.1 ± 10.9	59.5 ± 10.8	58.4 ± 11.0
*Cardiometabolic*			
Metabolic Syndrome score	−8.2 ± 2.7	−8.3 ± 2.8	−7.9 ± 2.5
CRP*	0.63 ± 0.99	0.62 ± 1.0	0.65 ± 0.81
Systolic blood pressure	112 ± 10	112 ± 10	111 ± 10
Diastolic blood pressure	72 ± 7	72 ± 8	71 ± 7
Total cholesterol: HDL ratio*	3.5 ± 1.6	3.5 ± 1.5	3.5 ± 1.4
Icam1	165 ± 40	165 ± 43	165 ± 34
Il1b*	0.38 ± 0.35	0.38 ± 0.29	0.33 ± 0.40
Il6*	1.52 ± 1.23	1.39 ± 1.29	1.65 ± 1.23
Il8*	1.49 ± 1.06	1.54 ± 1.00	1.38 ± 1.38
Leptin*	13,835 ± 16,792	14,801 ± 16,076	12,837 ± 17,173
Fasting glucose	82.5 ± 5.9	81.9 ± 5.8	83.6 ± 6.1
TNFA*	3.2 ± 1.6	3.2 ± 1.6	3.1 ± 1.6
Fasting insulin*	5.1 ± 3.3	5.1 ± 3.0	5.3 ± 3.5
AUC glucose	260.4 ± 41.8	260.1 ± 40.8	260.8 ± 43.7
AUC insulin*	87.3 ± 48.8	88.9 ± 45.4	80.1 ± 51.6
HOMAIR*	1.05 ± 0.67	1.0 ± 0.62	1.1 ± 0.87
HOMABeta*	93.1 ± 62.8	92.6 ± 59.7	94.2 ± 65.1
Insulin response*	0.79 ± 0.49	0.80 ± 0.47	0.75 ± 0.62
Insulin sensitivity*	0.19 ± 0.12	0.12 ± 0.12	0.19 ± 0.12

^a^ Data are presented as mean ± standard deviation for continuous variables and *n* (%) for categorical variables. Data presented as median ± IQR are indicated by an asterisk.

On average, at baseline, the CR group consumed approximately 47% of reported energy from carbohydrate, 34% from fat, and 17% from protein. Comparable results were observed in the control (45%, 35%, and 17%, respectively). The two randomized groups did not differ with respect to aMED scores (4.4 ± 1.8 and 4.0 ± 1.9 for CR and control, respectively), the DII (−0.29 ± 1.8 and 0.10 ± 1.7, respectively), or the HEI (59.5 ± 10.8 and 58.4 ± 11.0, respectively).

The average MSS was −8.2, with no difference between the CR and control (−8.3 ± 2.8 and −7.9 ± 2.5). Similar results were observed for CRP measurements at baseline (0.62 vs. 0.65). The two groups were comparable with respect to all other cardiometabolic measures (Table 1).

Measured and self‐reported energy intake values were compared for all participants (i.e., combined CR and control) at baseline, 12‐ and 24‐month visits and all differences were less than 20% on average. On average, measured values were greater than self‐reported values (means: 14.0%, 14.6%, and 15.7% at baseline, 12‐ and 24‐month visits, respectively; and medians: 16.0%, 15.0%, 16.0%, respectively).

### 3.2 Self‐selected diet composition changes during CR

**FIGURE 1:**
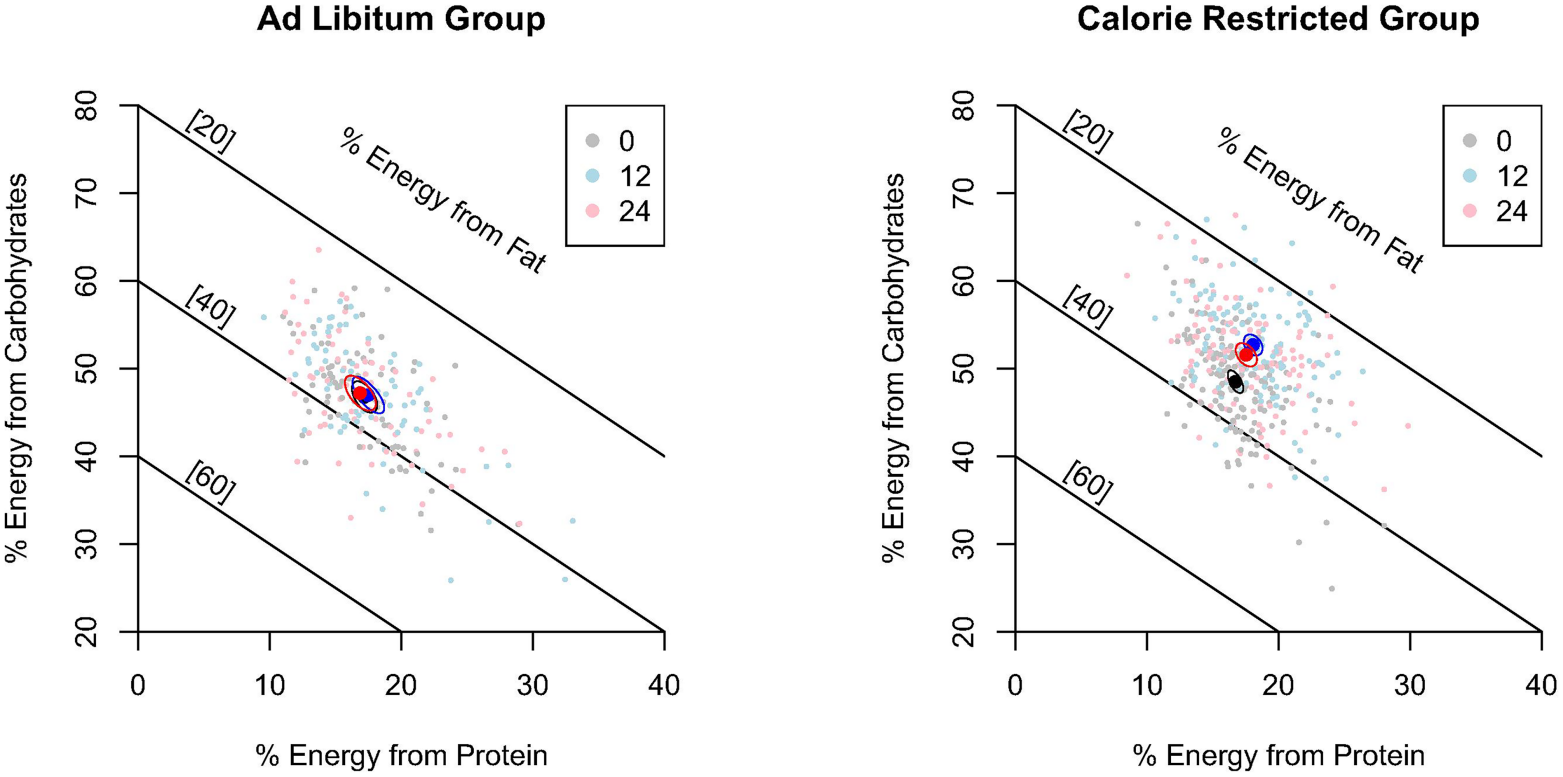
Right‐angle mixture triangle (RMT) of percentage reported energy from protein (*X*‐axis), carbohydrate (*Y*‐axis), and fat (implicit axis) in each group. Black, blue, and red are for baseline, 12‐ and 24‐month visits. Washed out underlying colors are raw data, and sharp colors, the mean. The ellipse is a 95% credible interval. Diagonal black lines are iso‐fat lines.

Changes in the macronutrient composition of the self‐selected diet for the CR and control groups are shown in Figure 1. The mean percentage reported energy from carbohydrate, fat, and protein at each study visit and average changes in reported energy intake for each macronutrient over time are reported in Table 2. Average scores and changes over time for the aMED, DII, and HEI are also reported. Changes in estimated logit proportions and 95% credible intervals for each macronutrient from baseline to 12 and 24 months are reported in Table 3 for each group. Intermediate time points (i.e., 6 and 18 months) are reported for the CR group only. We observed a statistically significant interaction between treatment and time on dietary composition, indicating that randomization to the CR intervention results in dietary changes that are significantly different from those observed in the control group at 12 and 24 months. Among control participants, no statistically significant changes in dietary composition were observed from baseline to 12 or 24 months. However, the CR participants had a significantly higher consumption of protein (logit proportion: 0.09; 95% credible interval: 0.05, 0.13) and carbohydrate (0.16; 0.12, 0.20) from baseline to 12 months during the intervention. These increases were accompanied by a statistically significant decrease in dietary fat (−0.18; −0.23, −0.12). For the CR group, the changes in dietary macronutrient composition at 24 months relative to baseline were comparable to the 12‐month changes observed in this group.

**TABLE 2:** Mean percentage reported energy intake from each macronutrient and mean dietary pattern index scores at each time point by randomized intervention group.

	Baseline^a^	12 months	24 months
*Calorie restriction*			
Carbohydrate	46.8 ± 6.5	50.4 ± 5.8	49.2 ± 6.2
Δ from baseline	–	3.5 ± 6.6	2.3 ± 6.5
Fat	33.5 ± 4.9	28.9 ± 5.1	30.2 ± 5.1
Δ from baseline	–	−4.4 ± 6.4	−3.1 ± 6.1
Protein	16.6 ± 3.0	18.1 ± 3.4	17.5 ± 3.7
Δ from baseline	–	1.6 ± 3.6	1.0 ± 3.8
Alternate Mediterranean Diet Index	4.4 ± 1.8	5.0 ± 1.7	5.1 ± 1.9
Δ from baseline	–	0.6 ± 2.0	0.6 ± 1.9
Dietary Inflammatory Index	−0.29 ± 1.84	−1.33 ± 1.85	−1.08 ± 2.03
Δ from baseline	–	−0.98 ± 1.68	−0.70 ± 1.65
Healthy Eating Index	59.5 ± 10.8	67.9 ± 10.4	67.5 ± 12.2
Δ from baseline	–	8.2 ± 11.6	7.6 ± 11.9
*Ad libitum control*			
Carbohydrate	45.1 ± 6.3	45.4 ± 7.1	45.1 ± 6.7
Δ from baseline	–	0.2 ± 6.5	0.2 ± 6.4
Fat	34.7 ± 4.9	34.2 ± 4.9	34.5 ± 5.6
Δ from baseline	–	−0.6 ± 5.1	−0.4 ± 6.2
Protein	17.2 ± 3.5	17.6 ± 4.4	16.9 ± 4.3
Δ from baseline	–	0.5 ± 3.7	−0.2 ± 3.3
Alternate Mediterranean Diet Index	4.0 ± 1.9	4.1 ± 2.0	4.0 ± 1.9
Δ from baseline	–	0.01 ± 2.0	0.04 ± 1.9
Dietary Inflammatory Index	0.10 ± 1.71	−0.03 ± 1.91	−0.23 ± 1.99
Δ from baseline	–	−0.10 ± 1.35	−0.39 ± 1.34
Healthy Eating Index	58.4 ± 11.0	58.6 ± 11.9	59.1 ± 11.8
Δ from baseline	–	0.1 ± 12.4	1.3 ± 11.4

^a^ Data are presented as mean ± standard deviation.

**TABLE 3:** Estimated proportion of reported energy from each macronutrient at baseline and over time by randomized intervention group.

	Calorie restriction^a^	Sig _CR_ ^b^	Ad libitum control^a^	Sig _AL_ ^b^	Sig _CR‐AL_ ^c^
Baseline					
Protein	−1.61 (−1.65, −1.58)	–	−1.58 (−1.64, −1.52)	–	–
Carbohydrate	−0.06 (−0.10, −0.02)	–	−0.14 (−0.20, −0.07)	–	–
Fat	−0.64 (−0.68, −0.60)	–	−0.58 (−0.63, −0.53)	–	–
6‐month change from baseline					
Protein	0.09 (0.05, 0.12)	*	–	–	–
Carbohydrate	0.14 (0.09, 0.18)	*	–	–	–
Fat	−0.22 (−0.27, −0.17)	*	–	–	–
12‐month change from baseline					
Protein	0.09 (0.05, 0.13)	*	0.02 (−0.04, 0.08)	NS	*
Carbohydrate	0.16 (0.12, 0.20)	*	0.01 (−0.06, 0.07)	NS	*
Fat	−0.25 (−0.30, −0.20)	*	−0.02 (−0.09, 0.04)	NS	*
18‐month change from baseline					
Protein	0.10 (0.06, 0.14)	*	–	–	–
Carbohydrate	0.11 (0.07, 0.16)	*	–	–	–
Fat	−0.20 (−0.25, −0.16)	*	–	–	–
24‐month change from baseline					
Protein	0.06 (0.02, 0.10)	*	−0.02 (−0.07, 0.04)	NS	*
Carbohydrate	0.11 (0.07, 0.16)	*	0.02 (−0.05, 0.08)	NS	*
Fat	−0.18 (−0.23, −0.12)	*	−0.01 (−0.07, 0.05)	NS	*

^a^ Data are presented as estimated logit proportions (95% credible intervals) derived from an MR‐GLMM.

^b^ Significance of changes over time within‐group (CR and AL separately). Effects are considered significant when the estimated difference has a credible interval that does not span 0. Significant effects are denoted by an asterisk (*).

^c^ Significance of changes over time between groups (CR vs. AL). Effects are considered significant when the estimated difference has a credible interval that does not span 0. Significant effects are denoted by an asterisk (*).

### 3.3 Self‐selected diet composition changes as predictors of short‐ (12 months) and long‐term (24 months) adherence to CR

**FIGURE 2:**
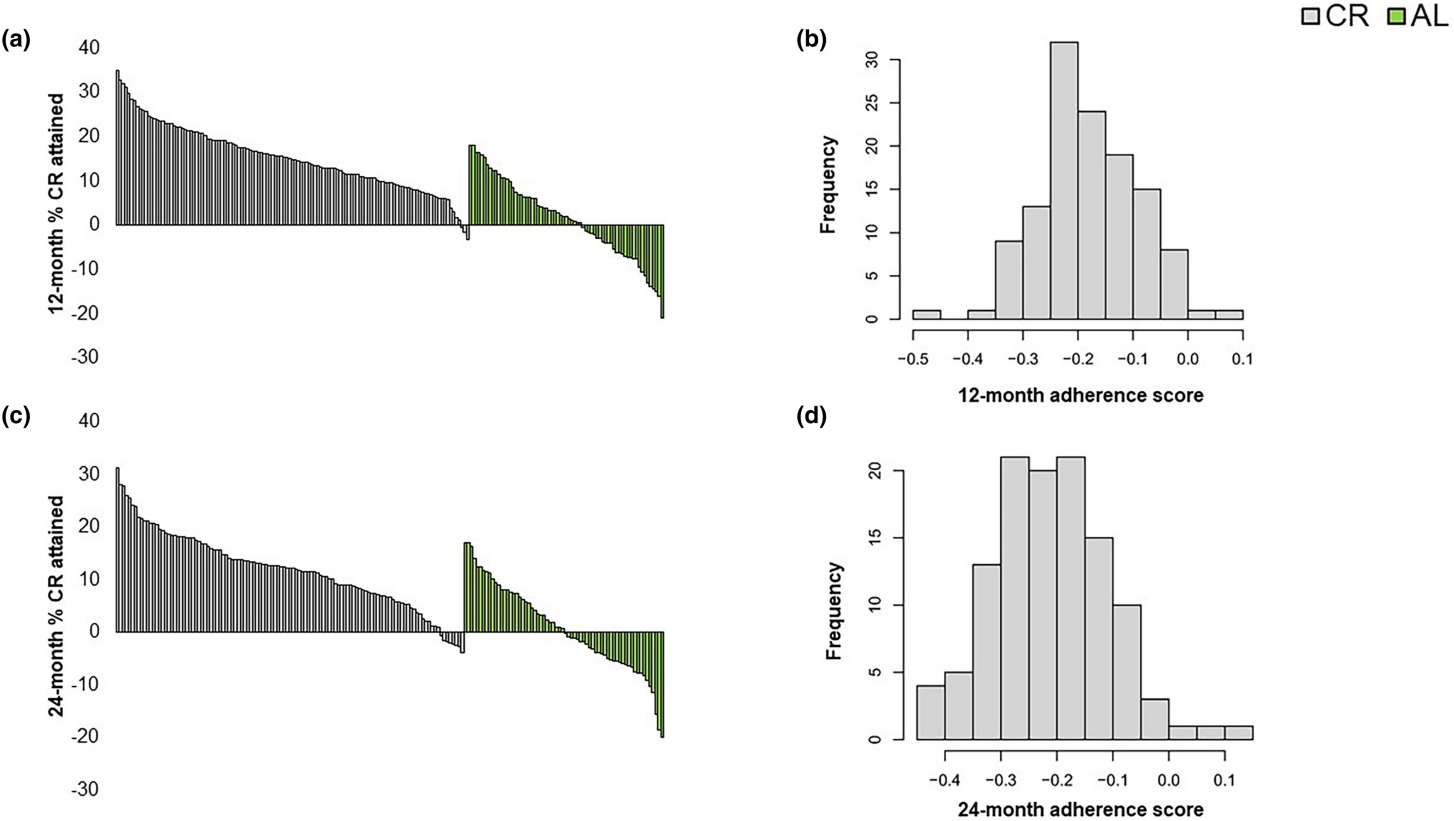
Distribution of adherence to calorie restriction (CR) at 12 (a, b) and 24‐month follow‐up visits (c, d) in the CR and ad libitum (AL) control groups. Percent calorie restriction (a, c) is shown with CR in gray and AL in green. Adherence for the CR participants are shown in (b, d), with adherence calculated as the natural log of the ratio of prescribed energy intake to total energy intake.

The variability in CR adherence is shown in Figure 2. At 12 months, the level of CR attained ranged from −3% (i.e., consuming more energy than was prescribed) to 35% (i.e., consuming lesser energy than was prescribed or a greater than prescribed CR) in the intervention group and from −21% to 18% among control participants (Figure 2a). Median adherence at 12 months was 14.8% in the CR group and 1.9% among controls. Among CR participants only at 12 months (Figure 2b), reported energy intake was higher than the prescribed CR. Comparable results within each group were observed at 24 months relative to baseline, with ranges of adherence from −4% to 31% and −20% to 18% in CR (median: 12.2%) and control (median: 0.3%), respectively (Figure 2c). Approximately 9% of CR participants achieved the 25% CR benchmark at 12 months, with only 4% at 24 months. In the CR group, more negative adherence score values are observed at 24 months (Figure 2d) compared to 12 months (Figure 2b), indicating that participants were consuming more calories than prescribed and adherence to the intervention tended to decrease over time.

**FIGURE 3:**
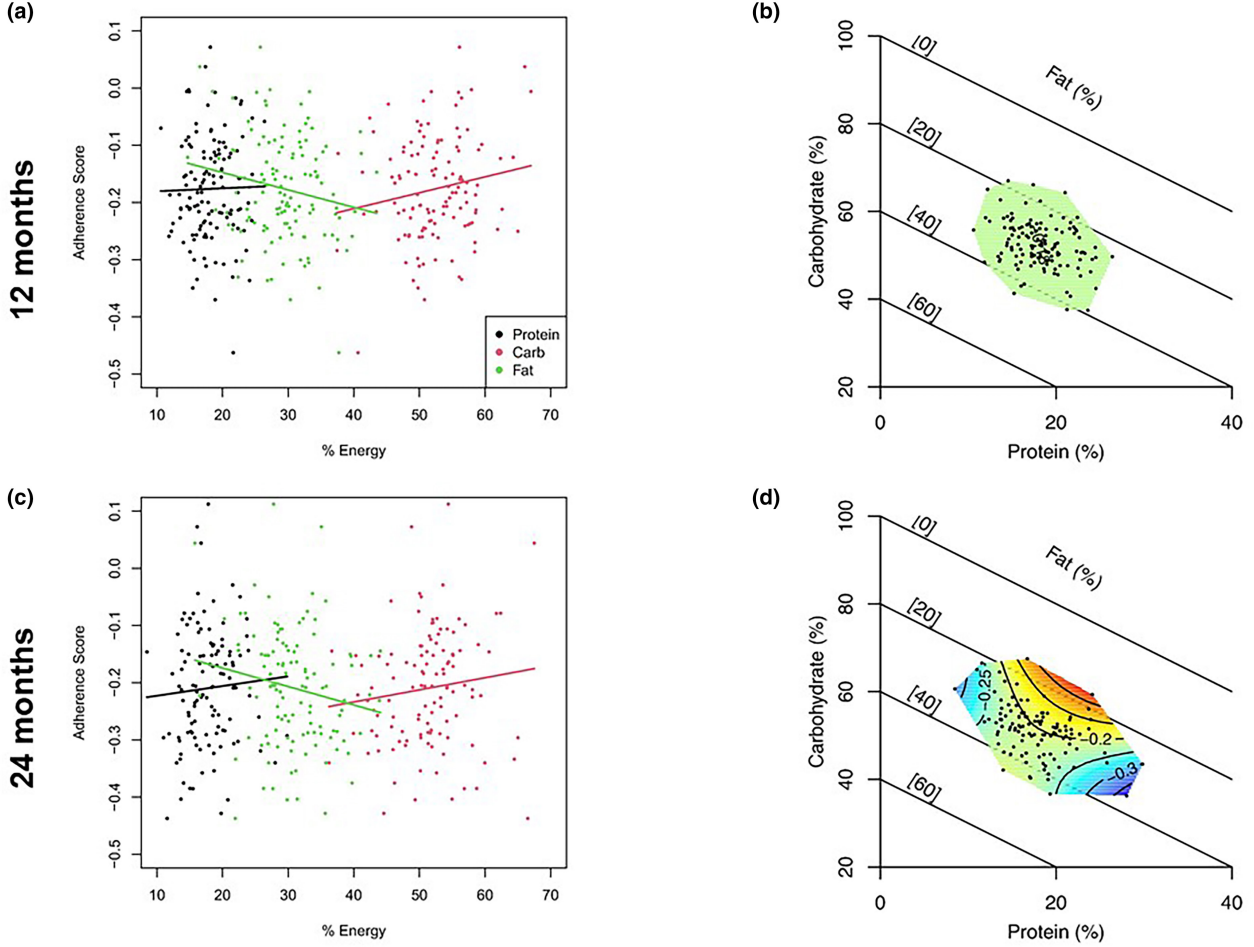
Association between percentage reported energy from carbohydrate, fat, and protein at 12‐ and 24‐month follow up visits (a, c). Fitted trends are from linear regression to help interpret the sign of the association (coefficients not shown). Right‐angle mixture triangles at each time point (b, d) show the association between percentage reported energy from each macronutrient and adherence. Surface is as estimated by the AIC‐favored model. Red areas indicate high adherence, while blue areas indicate low adherence. Contours indicate the absolute value of adherence score.

We used mixture models to assess whether dietary composition affected adherence to CR. At the 12‐month time point, a null model was favored by AIC (AIC_null_ = −241, AIC_linear_ = −241, AIC_quadratic_ = −236), indicating that diet composition was not a strong predictor of adherence at 12 months of CR. Additionally, there was little independent association between percentage protein consumption and adherence. However, at 24 months, the quadratic mixture model was favored indicating a nonlinear effect of macronutrients (AIC_null_ = −195, AIC_linear_ = −194, AIC_quadratic_ = −197). Specifically, protein in the background of carbohydrate and fat consumption was associated with differential adherence to the intervention. More specifically, consuming a diet with above 18% protein and 55% carbohydrate and below 30% fat had the highest adherence scores. Furthermore, on a relatively high carbohydrate diet (e.g., approximately 60%), substituting protein for fat resulted in the largest gains in adherence during the intervention, whereas on a more moderate carbohydrate diet (e.g., approximately 45%), substituting protein for fat results in lower adherence. These results are visualized in Figure 3a–d. There was no association between diet pattern indices and adherence to CR (data not shown).

### 3.4 Association between macronutrient composition, dietary patterns, and cardiometabolic risk factors

Correlations between macronutrient consumption and cardiometabolic risk factor measurements at each time point are shown in the Table S1. The observed correlations were weak, with most ranging in strength from −0.3 to 0.3. The mixture‐model approach revealed no consistent effects of self‐selected diet composition on several highly correlated indicators of cardiometabolic health in the CR group. However, statistically significant correlations between risk factors and dietary pattern indices were observed. In particular, CRP was positively correlated with a more pro‐inflammatory dietary pattern (i.e., a higher DII score) at 12 and 24 months (ρ = 0.29; *p* < 0.001 and ρ = 0.25; *p* = 0.007, respectively). Consistent results for higher CRP were observed with lower adherence to the Dietary Guidelines for Americans (i.e., a lower HEI score) at 12 months (ρ = −0.20; *p* = 0.03); however, statistical significance was not retained at 24 months (ρ = −0.09; *p* = 0.36). There was also an inverse relationship between metabolic syndrome and the HEI (ρ = −0.20; *p* = 0.03). Statistically significant correlations of the aMED, DII, and HEI with glucoregulatory markers and leptin were also observed.

## 4 DISCUSSION

Research using the GFN suggests that the benefits of CR generated by dietary dilution in ad libitum fed animals cannot be attributed solely to a reduction in calories. These studies indicate that the ratio of dietary macronutrients also plays an important role in reducing age‐related disease in animals, with potential implications in human populations (Ingram & de Cabo, 2017; Rizza et al., 2014; Solon‐Biet et al., 2014). In particular, in animal models, a higher protein‐to‐carbohydrate ratio has been associated with impaired metabolic health and accelerated aging (Le Couteur et al., 2016; Lee et al., 2015; Solon‐Biet et al., 2015). This finding is consistent with the lower protein, higher carbohydrate dietary patterns observed among healthy, longer living human populations (Le Couteur et al., 2016; Senior et al., 2020; Willcox et al., 2009). In contrast, randomized controlled trials conducted in humans (Gosby et al., 2011, 2014) report that high dietary protein content is associated with increased satiety and reduced food intake, suggesting that dietary protein may enhance adherence to CR. In addition, it remains unclear whether dietary dilution produces benefits for aging; studies have shown that life span is reduced in mice where dietary dilution achieves decreases in calorie intake compared to standard calorie restriction approaches (Pak et al., 2021; Solon‐Biet et al., 2014). The complexities associated with conducting nutrition trials in humans preclude the implementation of a fully factorial GFN trial similar to those performed in animals where multiple simultaneous manipulations and substitutions of macronutrient composition can be implemented (Solon‐Biet et al., 2015). The present study is unique in that it uses the GFN with calorie restriction achieved without diet dilution.

To our knowledge, this is the first analysis linking dietary macronutrient composition in relation to adherence to the CR prescription and to cardiometabolic risk in humans using the state‐of‐the‐art GFN approach in a population of calorically restricted humans without obesity. All previous applications of the GFN have focused on either animal models or humans with ad libitum access to food. The GFN aims to discern the complex relationships among nutrients and their combined effects on chronic disease risk reduction (Raubenheimer & Simpson, 2016; Simpson, Le Couteur, James et al., 2017; Simpson, Le Couteur, Raubenheimer et al., 2017). As the GFN method considers the synergistic interactions among nutrients and their impact on disease, this approach overcomes inherent limitations of the “one nutrient at a time” analytical paradigm, which is associated with inflated rates of type‐1 error, as well as missing potentially important synergistic interactions between nutrients (Raubenheimer & Simpson, 2016).

Our findings suggest that calorie restriction results in alterations in macronutrient intake with a shift towards greater consumption of protein and carbohydrate and lower fat intake. These shifts in macronutrient intake, however, were not observed in the control group and one could argue that the adaptation to sustaining a CR regimen may have required an alteration in the diet composition. That said, although participants were not provided food or prescribed a specific diet pattern, they had access to diet counselors who provided broad dietary guidance to help facilitate CR adherence, and it is likely that such guidance was supportive of greater protein intake and lower fat intake combined with average daily macronutrient recommendations for carbohydrate, and this guidance may have influenced the observed changes in intake. Furthermore, dietary protein was not an independent contributor to CR adherence both in the short term (at 12 months) and over the longer term (at 24 months). The diet composition that supported the best long‐term adherence was higher than average in protein consumption in the background of higher carbohydrate and lower fat intake. On a relatively high carbohydrate diet (~60%), substituting protein for fat led to big gains in adherence whereas on a more moderate carbohydrate diet (~45%), substituting protein for fat resulted in lower adherence. These findings underscore the complex interplay between macronutrients and suggest that perhaps the role of carbohydrate intake in adherence to CR may warrant further investigation. Future research involving macronutrient substitutions is required to definitively conclude the impact of these observations on long‐term adherence.

Epidemiologic studies consistently show that individual nutrients, foods, and food groups are associated with the onset or progression of some chronic diseases. However, despite marked improvements in cardiometabolic outcomes in CALERIE (Kraus et al., 2019), macronutrient composition was not consistently associated with reductions in cardiometabolic risk factors in this group of humans without obesity. These observations extend to protein intake, which was not positively or negatively linked with cardiometabolic outcomes. The absence of adverse impact of dietary protein on cardiometabolic outcomes is encouraging in the light of previous findings linking dietary protein restriction or low protein intake with improvements in cardiometabolic risk factors and markers of disease risk and overall mortality in humans (Levine et al., 2014; Treviño‐Villarreal et al., 2018).

It is important to note that in this study, individual macronutrients were not indicative of strong systemic effect on cardiometabolic outcomes. However, foods and nutrients are consumed in combination and have synergistic effects on health. In our analyses, overall dietary patterns were assessed using three widely used indices that capture different components of a healthy diet and revealed consistent short‐ and longer term relationships with cardiometabolic outcomes in this group of individuals without obesity and without chronic disease. Diet‐quality scores are increasingly used in risk prediction for all‐cause mortality, cardiovascular disease, and other chronic diseases as an alternative to single‐food and single‐nutrient approaches (Hodge et al., 2018; Lassale et al., 2016; Shivappa et al., 2018; Silver et al., 2023) and these scores have been applied previously in the CALERIE trial with respect to cognitive function (Silver et al., 2023). Our results underscore the importance of investigating the impact of overall dietary patterns on health outcomes rather than simply examining one macronutrient at a time.

Strengths of this study include the highly defined and rigorously collected CALERIE data, including for self‐selected diet composition, objectively measured CR, and comprehensive measures of health outcomes. Furthermore, the synergistic effects of dietary determinants on cardiometabolic risk during CR were determined by applying the GFN approach to CALERIE data which allows for the simultaneous examination of multiple nutrients. The diet data nevertheless are self‐reported, and is a limitation which is somewhat mitigated by the high quality of the reported intake in comparison to the objectively measured doubly labeled water assessment of actual caloric intake. At each time point, the average difference in self‐reported energy intake and measured energy intake was well within ±20% (i.e., 14% at baseline, 14.6% at 12 months, and 15.7% at 24 months).

Our findings are directly relevant to the design of future dietary interventions aimed at optimizing the benefits associated with CR. In addition, our analysis lends support for the use of GFN as an analytical paradigm for similar data collected in other populations, including in individuals with overweight or obesity in whom the role of diet composition during CR for the promotion of adherence and reducing cardiometabolic risk may likely be more important.

In conclusion, in this study of individuals without obesity, CR resulted in a shift toward greater protein and carbohydrate intake; higher protein in the background of high carbohydrate intake was a predictor of higher adherence to CR. Our analyses suggest that dietary patterns, and not individual macronutrients per se, have an impact on cardiometabolic risk in healthy adults without obesity. Further research involving macronutrient manipulations or substitutions is required to examine the impact of intervention‐related impact of macronutrients in humans with and without obesity.

## AUTHOR CONTRIBUTIONS

Sai Krupa Das, Rachel E. Silver, Alistair Senior, and David Le Couteur designed the research objectives and analyses. Alistair Senior and Rachel E. Silver conducted the statistical analyses. Sai Krupa Das, Rachel E. Silver, and Alistair Senior interpreted the results. Sai Krupa Das and Rachel E. Silver wrote the manuscript with a critical review from all coauthors. All authors read and approved the final manuscript.

## FUNDING INFORMATION

This work was supported by the National Institutes of Aging  (R21AG064295‐02 SKD); Sai Krupa Das is also supported by NIA grant # R01AG071717‐02 and from the USDA Agricultural Research Service: 58‐8050‐4‐003 : 58‐8050‐9‐004.

## CONFLICT OF INTEREST STATEMENT

David Le Couteur is a founder of EndoAxiom which holds patents on drugs for the prevention and treatment of diabetes mellitus. All other authors have no conflicts of interest to disclose.

## Supporting information


Table S1.

## Data Availability

This secondary analysis used publicly available, de‐identified data from the CALERIE study data repository, available for download at https://calerie.duke.edu/.
