# Supplementary material for: Diet composition, adherence to calorie restriction, and cardiometabolic disease risk modification
Source: Aging Cell. 2023 Oct 24;22(12):e14018. doi: 10.1111/acel.14018 (PMC10726801; doi:10.1111/acel.14018)
Supplement: Supplementary file 1 — Table S1. [file ACEL-22-e14018-s001.docx]

**Supplementary Table.** Correlations between cardiometabolic risk factors and dietary composition and dietary pattern indices among CR participants at baseline, 12 months, and 24 months follow up.

|  | **Carbohydrate** | | **Fat** | | **Protein** | | **aMED** | | **DII** | | **HEI-2015** | |
| --- | --- | --- | --- | --- | --- | --- | --- | --- | --- | --- | --- | --- |
|  | **ρ** | ***P* _value_^1^** | **ρ** | ***P* _value_** | **ρ** | ***P* _value_** | **ρ** | ***P* _value_** | **ρ** | ***P* _value_** | **ρ** | ***P* _value_** |
| **C-reactive protein** |  |  |  |  |  |  |  |  |  |  |  |  |
| Baseline (n=143) | 0.05 | 0.55 | -0.06 | 0.47 | 0.07 | 0.41 | -0.10 | 0.25 | 0.16 | 0.06 | -0.12 | 0.16 |
| 12 months (n=126) | 0.08 | 0.37 | 0.01 | 0.90 | -0.07 | 0.46 | -0.17 | 0.06 | **0.29** | **<0.001** | **-0.20** | **0.03** |
| 24 months (n=118) | 0.09 | 0.36 | -0.18 | 0.05 | 0.10 | 0.25 | -0.10 | 0.29 | **0.25** | **0.007** | -0.09 | 0.36 |
| **Metabolic syndrome score** |  |  |  |  |  |  |  |  |  |  |  |  |
| Baseline (n=143) | 0.05 | 0.52 | 0.07 | 0.41 | 0.001 | 0.99 | -0.07 | 0.43 | -0.01 | 0.89 | **-0.18** | **0.03** |
| 12 months (n=126) | 0.04 | 0.69 | -0.10 | 0.28 | 0.17 | 0.06 | -0.05 | 0.61 | 0.11 | 0.21 | -0.08 | 0.40 |
| 24 months (n=118) | 0.13 | 0.18 | -0.11 | 0.23 | 0.10 | 0.30 | -0.17 | 0.07 | 0.11 | 0.25 | -0.04 | 0.70 |
| **ICAM1** |  |  |  |  |  |  |  |  |  |  |  |  |
| Baseline (n=142) | 0.13 | 0.14 | -0.03 | 0.71 | 0.02 | 0.81 | -0.07 | 0.39 | -0.01 | 0.94 | -0.06 | 0.50 |
| 12 months (n=125) | -0.02 | 0.85 | -0.03 | 0.77 | 0.07 | 0.43 | -0.04 | 0.62 | -0.03 | 0.78 | 0.03 | 0.72 |
| 24 months (n=118) | -0.06 | 0.51 | 0.13 | 0.15 | -0.11 | 0.23 | 0.03 | 0.72 | -0.04 | 0.64 | -0.08 | 0.42 |
| **Leptin** |  |  |  |  |  |  |  |  |  |  |  |  |
| Baseline (n=143) | 0.13 | 0.12 | -0.01 | 0.94 | -0.15 | 0.08 | -0.06 | 0.45 | **0.33** | **<0.001** | 0.06 | 0.45 |
| 12 months (n=126) | 0.12 | 0.17 | 0.02 | 0.84 | -0.05 | 0.58 | -0.11 | 0.20 | **0.38** | **<0.001** | -0.08 | 0.36 |
| 24 months (n=118) | 0.11 | 0.22 | 0.05 | 0.61 | -0.09 | 0.34 | **-0.21** | **0.02** | **0.39** | **<0.001** | -0.08 | 0.40 |
| **AUC Glucose** |  |  |  |  |  |  |  |  |  |  |  |  |
| Baseline (n=105) | -0.03 | 0.76 | -0.09 | 0.36 | 0.09 | 0.37 | -0.13 | 0.18 | 0.07 | 0.47 | -0.02 | 0.83 |
| 12 months (n=123) | -0.16 | 0.09 | 0.07 | 0.44 | 0.03 | 0.71 | 0.02 | 0.78 | 0.09 | 0.32 | -0.02 | 0.83 |
| 24 months (n=116) | -0.01 | 0.93 | -0.14 | 0.14 | 0.10 | 0.28 | 0.02 | 0.87 | 0.03 | 0.76 | 0.03 | 0.73 |
| **AUC Insulin** |  |  |  |  |  |  |  |  |  |  |  |  |
| Baseline (n=96) | -0.11 | 0.28 | 0.19 | 0.06 | 0.05 | 0.65 | **-0.30** | **0.003** | **0.29** | **0.004** | **-0.31** | **0.002** |
| 12 months (n=109) | 0.07 | 0.49 | 0.03 | 0.78 | -0.10 | 0.30 | **-0.20** | **0.03** | **0.27** | **0.004** | -0.15 | 0.13 |
| 24 months (n=116) | 0.01 | 0.94 | 0.05 | 0.56 | -0.04 | 0.71 | **-0.37** | **<0.001** | **0.36** | **<0.001** | -0.15 | 0.10 |
| **HOMA-IR** |  |  |  |  |  |  |  |  |  |  |  |  |
| Baseline (n=143) | -0.09 | 0.28 | **0.19** | **0.03** | 0.08 | 0.32 | **-0.34** | **<0.001** | **0.21** | **0.01** | **-0.30** | **<0.001** |
| 12 months (n=126) | -0.02 | 0.87 | 0.12 | 0.19 | -0.01 | 0.94 | **-0.19** | **0.04** | **0.20** | **0.03** | -0.13 | 0.14 |
| 24 months (n=118) | -0.04 | 0.69 | 0.09 | 0.33 | 0.02 | 0.79 | **-0.34** | **<0.001** | **0.31** | **<0.001** | **-0.17** | **0.06** |
| **HOMA-Beta** |  |  |  |  |  |  |  |  |  |  |  |  |
| Baseline (n=143) | -0.02 | 0.78 | 0.13 | 0.11 | 0.02 | 0.77 | **-0.35** | **<0.001** | **0.38** | **<0.001** | **-0.30** | **<0.001** |
| 12 months (n=126) | 0.06 | 0.47 | 0.12 | 0.17 | -0.07 | 0.42 | **-0.22** | **0.01** | **0.26** | **0.003** | -0.08 | 0.40 |
| 24 months (n=118) | 0.10 | 0.28 | 0.04 | 0.66 | -0.11 | 0.24 | **-0.33** | **<0.001** | **0.36** | **<0.001** | -0.12 | 0.21 |
| **Insulin response** |  |  |  |  |  |  |  |  |  |  |  |  |
| Baseline (n=99) | -0.10 | 0.34 | **0.29** | **0.003** | -0.04 | 0.72 | -0.10 | 0.31 | 0.18 | 0.07 | -0.20 | 0.05 |
| 12 months (n=115) | **0.21** | **0.02** | -0.12 | 0.21 | -0.08 | 0.41 | -0.15 | 0.12 | 0.13 | 0.17 | -0.05 | 0.58 |
| 24 months (n=116) | -0.01 | 0.95 | 0.10 | 0.26 | 0.04 | 0.70 | **-0.28** | **0.002** | **0.25** | **0.008** | **-0.20** | **0.03** |
| **Insulin sensitivity** |  |  |  |  |  |  |  |  |  |  |  |  |
| Baseline (n=143) | 0.08 | 0.33 | **-0.18** | **0.03** | -0.08 | 0.37 | **0.36** | **<0.001** | **-0.26** | **0.002** | **0.32** | **<0.001** |
| 12 months (n=126) | -0.01 | 0.94 | -0.12 | 0.19 | 0.02 | 0.80 | **0.20** | **0.03** | **-0.21** | **0.02** | 0.11 | 0.21 |
| 24 months (n=118) | 0.01 | 0.90 | -0.09 | 0.35 | -0.003 | 0.98 | **0.35** | **<0.001** | **-0.34** | **<0.001** | 0.18 | 0.06 |

^1^Calculated for Spearman’s rank correlations, with statistically significant correlations are shown in bold in order to facilitate interpretation of results.
